# Colonic Metastases From Lung Carcinoma: A Case Report and Review of the Literature

**DOI:** 10.4021/gr518e

**Published:** 2013-03-09

**Authors:** Ana Isabel Gonzalez-Tallon, Jorge Vasquez-Guerrero, Maria Angeles Garcia-Mayor

**Affiliations:** a Gastroenterology Department, Central Hospital of Defense “Gomez Ulla”, Madrid, Spain

**Keywords:** Colon carcinoma, Lung carcinoma metastasis, Colonic metastasis, Gut metastasis, Small bowel metastasis, Gastrointestinal metastasis, Lung carcinoma, Colorectal metastasis, Rectorrhagia, Gastrointestinal bleeding, Gut bleeding

## Abstract

Lung cancer is the most frequent cause of cancer death in the world. Although about 50% of lung cancers have distant metastases at the time of diagnosis, gastrointestinal metastasis has rarely been described. The most common metastatic site is the small bowel, whereas, colonic metastases are very rare. This report presents a clinical case of a 68-year-old male with a previous diagnosis of non-microcytic lung carcinoma (T4, N2, M1), stage IV, who presented rectorrhagia at the emergency. Colonoscopy showed many ulcerated tumors along the colon and histology proved that these lesions were metastases of primitive lung carcinoma. Gut metastasis from the lung is uncommon but we have to be aware of it in patients who present gastrointestinal symptoms.

## Introduction

Lung cancer is the most frequent cause of cancer death in the world [1]. About 50% of all lung cancers have distant metastasis at the time of initial diagnosis. Brain, liver, adrenal glands, and bone marrow are the most likely sites of metastatic disease in patients with lung cancer [2]. Metastases of the gastrointestinal tract are very uncommon. We report a case of colon metastases from lung carcinoma presenting with rectorrhagia as the first manifestation of gut metastasis.

## Case Report

Male patient, 68 years old, with history of surgery, because of perforated gastric ulcer about 40 years ago. Ex-smoker was for one year with pack cigarette index/year of 50. He arrived at the emergency room on July 2012 with rectal bleeding but conserving hemodynamic stability. There were no other symptoms such as: abdominal pain or obstruction data. He was diagnosed with non-microcytic cell carcinoma of the lung (TNM: T4, N2, M1), stage IV since February 2012. The thorax computer tomography (CT) had showed a lung mass in the left lower lobe with associated pneumonitis, parahiliar and cervical adenopathies and one metastasis in the suprarenal right gland. The same was shown in the positron emission tomography (PET-CT), he had no other metastasis in the body. Carcinoma immunostaining was positive for CK7, and negative for CK5-6, p63 and CD56. He received treatment with oncologic therapy with Cisplatin - Pemetrexed three times per week, radiotherapy and corticosteroids. He had been admitted previously to the hospital for malignant fever caused by the tumor, low respiratory tract infections and mucositis. Physical examination did not show any relevant alteration with the exception of rectal bleeding in the rectal examination.

He had normocytic normocrhomic anemia diagnosed since January 2012, with hemoglobin values between 7.9 and 9.1 g/dL, but without evidence until the actual onset of macroscopic bleeding. Lab values a the onset were: Hemoglobin 8.5 g/dL, mean cell volume (MCV): 89.9 fl, mean cell hemoglobin (MCH): 28.5 pg, glucose: 104 mg/dL, Urea: 48 mg/dL, creatinine: 1.24 mg/dL, aspartate aminotransferase (AST): 38 U/L, alanine aminotransferase (ALT): 36 U/L, total bilirubin: 0.3 mg/dL, amylase: 33 U/L; sodium: 134 mEq/L, potassium: 4.4 mEq/L, C-reactive protein (CRP): 47.26 mg/dL, prothrombin time (PT): 73.77%, white blood cells: 53,140/mm^3^ (Neutrophils: 79%), platelets: 230,000/mm^3^.

**Figure 1:**
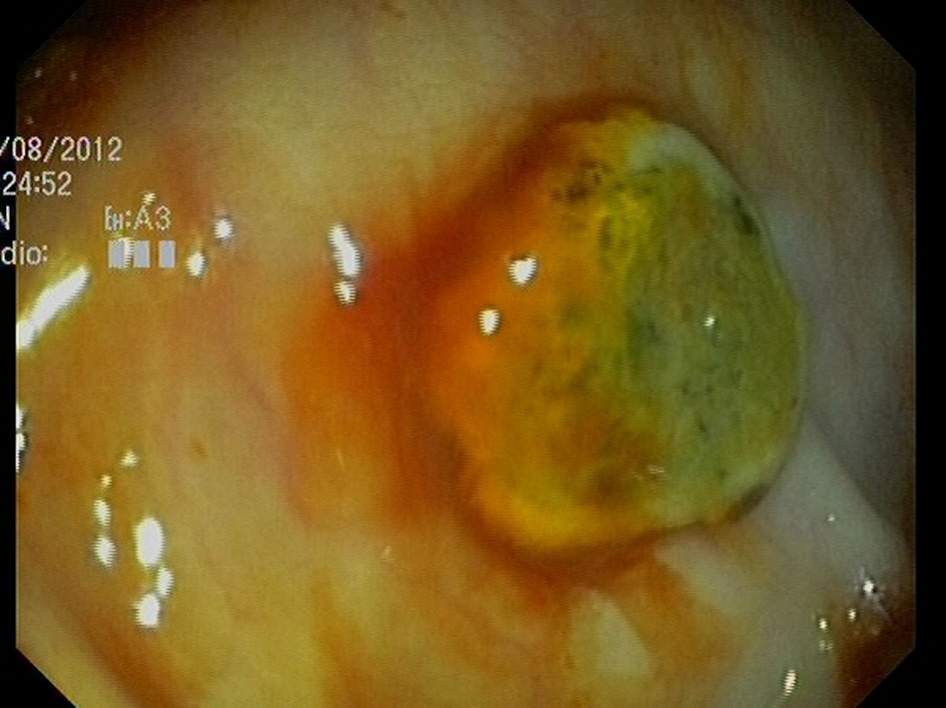
Lesion with button-like appearance, raised, smooth edges and ulcerated in the center, with biopsies compatible with lung carcinoma metastases.

**Figure 2:**
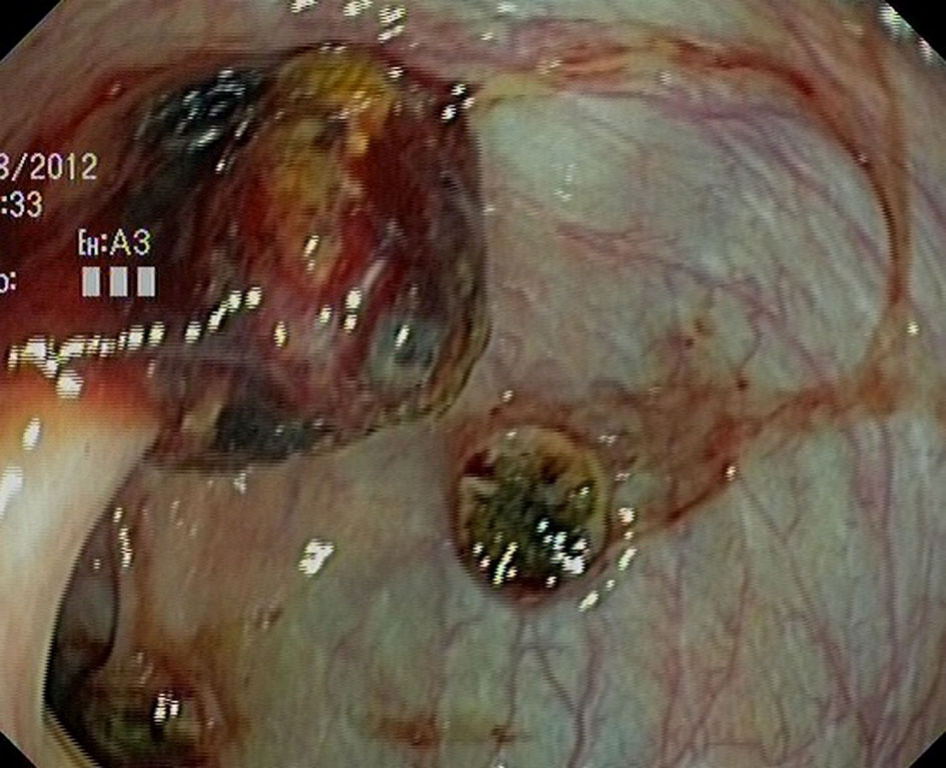
Colonic metastasis from the lung. One of them, probably, on a prior colonic polyp.

Abdominal radiography did not show dilated bowel loops or fluid levels, with adequate distribution of intestinal gas. Gastroscopy (08/08/12) did not find hematic residue or potential bleeding lesion. Colonoscopy (09/08/12), to the cecum, found many ulcerated tumors in the cecum, transverse and descendent colon with signs of recent bleeding (Fig. 1, 2).

The pathological report of the colonic biopsies was non-microcytic solid undifferentiated malignant tumor with CD 117 negative, Actin negative, Epithelial membrane antigen (EMA) negative, CK pool (AE1/AE3) moderate positivity, Ki 67 high positivity, DOG -1 negative, CD 34 negative, Desmin negative, compatible with lung tumor’s metastasis.

Because of clinical situation palliative surgery was dismissed and patient was handled conservatively.

## Discussion

Metastases of lung cancer to the digestive tract are uncommon, mostly asymptomatic and occur in patients with terminal stage disease. The actual incidence of these metastases is not really known. Most publications in the literature are isolated cases and reviewed retrospectively. Kim et al. revealed that gastrointestinal metastases were detected in 10 (0.19%) of 5,239 lung cancer patients [3]. The reported incidence of gastrointestinal metastases by Ryo M et al. in a study with 1,635 patients was 1.8%; 0.4% to the stomach, 1.1% to the small intestine and 0.5% to the colon [4]; In a more recent publication Yang et al [5] found 6 gastrointestinal metastases in a group of 339 patients with lung cancer (1.77%). One of them was cecal metastasis.

The publication of McNeill that found an average of 4.8 metastatic sites in small bowel [6] is interesting.

All these data demonstrate that the incidence of lung cancer metastases to the gastrointestinal tract is higher that is clinically apparent and more frequent in necropsies series [4, 7].

Gastrointestinal metastases have probably been underdiagnosed in living patients because their symptoms and signs are considered to be side effects of chemotherapy such colitis, ulcers, enteritis.

The reported incidence of symptomatic small intestine metastasis is 0.2-0.5% [8]. Esophagus is the more frequent gastrointestinal metastatic site, by contiguity invasion and small bowel and stomach by hematogenous dissemination [9].

From 1978 to the present we have found about 40 cases of colon metastases from the lung published in the literature [4, 5, 9-33], showed in the next table (Table 1).

**Table 1:** Forty Cases of Colon Metastases From the Lung Published in the Literature Since 1978 [4, 5, 9-33]

Author	Year	N cases	Symptoms	Lung carcinoma
Sakai H [10]	2012	1	Abdominal pain	Squamous cell carcinoma
Cedres S [9]	2012	1	Asymptomatic	Squamous cell carcinoma
Hsing CT [11]	2012	1	Acute abdominal pain	Adenocarcinoma
Fujiwara A [12]	2011	4	-	Non small cell lung cancer
Ceretti AP [13]	2011	1	Intestinal obstruction	Adenocarcinoma
Weng MW [14]	2010	1	Intestinal obstruction	Adenocarcinoma
Ahn SE [15]	2009	1	Anorexia	Adenocarcinoma
Hirasaki S [16]	2008	1	Asymptomatic	Squamous cell carcinoma
Ma Xt [17]	2008	1	Hypercalcemia	Squamous cell carcinoma
Goh BK [18]	2007	1	Abdominal pain	Large cell carcinoma
Yang CJ [5]	2006	1	Bloody stool	Small cell carcinoma
Stinchcombe TE [19]	2006	1	Asymptomatic	Squamous cell carcinoma
Uner A [20]	2005	1	-	-
Habesoglu MA [21]	2005	1	Intestinal obstruction	Squamous cell carcinoma
Jonh AK [22]	2002	1	Diarrhea	Undifferentiated large cell carcinoma.
Rouhanimanesh Y [23]	2001	1	Intestinal obstruction	Squamous cell carcinoma
Carroll D [24]	2001	1	Diarrhea and weight loss	Squamous cell carcinoma
Bastos I [25]	1998	1	Ileocolic Fistula	-
Ryo H [4]	1996	8	Asymptomatic (70%) Perforation Positive fecal blood test	Large cell carcinoma (3.7%) Adenocarcinoma (2.4%) Small cell carcinoma (1.7%) Squamous cell carcinoma 0.7%
Carr Sc [26]	1996	2	-	-
Johnson AO [27]	1995	1	Rectal bleeding	Small cell carcinoma
Gately CA [28]	1993	1	Rectorrhagia	Squamous cell carcinoma
Polak M [29]	1990	1	Perforation	Small cell carcinoma
Wegener M [30]	1988	1	Positive fecal blood test	Squamous cell carcinoma
Brown KL [31]	1980	1	-	Squamous cell carcinoma
Joffe N [32]	1978	2	Abdominal pain	Squamous cell carcinoma
Smith HJ [33]	1978	2	Intermittent obstruction Lower gastrointestinal tract bleeding	-

Colon metastases have been described with all kinds of lung carcinoma. But squamous cell carcinoma has been the one most reported [4, 19, 21, 24, 30-32]. However, with these data it is not possible to confirm a higher incidence of colonic metastasis in squamous cell carcinoma cases, compared with other lung carcinomas.

Patients with gastrointestinal metastasis of lung cancer are often asymptomatic [4]. The diagnosis of these metastases is usually a finding in the extension study [19, 34] (computer tomography or positron emission tomography). The diagnosis of about 1/3 of colonic metastases is made at autopsy [34].

The most common presenting symptoms are abdominal pain, and intestinal obstruction [4, 18, 21, 32]. Other symptoms are weight lost, bloody stools and diarrhea [4, 5, 22, 24]. However, there are not enough data to determine the true incidence of these symptoms.

Patients with digestive symptoms usually are in an advanced stage of their lung cancer. Lung cancer with intestinal metastasis has been reported to have a poor prognosis of less than 16 weeks in several studies [5, 21, 23].

Some of these reported patients have undergone surgery [12, 18, 35], but the conclusion of these studies is that aggressive surgical treatment is only worthwhile in a selected group. This conclusion is owing to the fact that some authors had discovered long-term survival, more than 2 years after surgery in patients with solitary metastasis [12], but in general, surgery only provides an effective palliation and is to be considered to prevent bowel obstruction or peritonitis.

Most of these studies are on patients with small bowel metastasis and conducted on very few patients [3, 12, 18, 35], so more studies are needed to determinate who really benefits from surgery.

Gastrointestinal metastases have been described more frequently like metachronous lesions in the context of lung cancer progression, but it can occur synchronously [9, 24].

It is noteworthy that some of the reported patients presented positive fecal blood test without data of macroscopic gastrointestinal bleeding [4, 5, 16, 23, 30]. This finding was the key to performing a diagnostic colonoscopy of colon metastasis.

Positive blood test has demonstrated good sensitivity, specificity and positive predicted value to detect advanced polyps and colon adenocarcinoma [36, 37]. This test is inexpensive compared with other diagnostics, principally imaging techniques (CT, PET-CT) and the cost effectiveness to detect colorectal carcinoma has been demonstrated [38, 39].

Perhaps, because of advanced improvement in chemotherapy, supportive care for lung cancer and extending life expectancy, we may come across an increasing number of gastrointestinal metastasis in the future.

Our patient underwent emergency colonoscopy because he presented an important rectorrhagia, where colon metastases from lung cancer were diagnosed. The colonoscopy showed multiple polypolidal lesions in the colon. Most reports showed a single colon tumor. In general, histological examination is the only way to make the diagnosis. Lung cancer involving the gastrointestinal tract usually mimics primary gastrointestinal tumors. So in order to distinguish primary gastrointestinal carcinoma from a metastasis of the lung the use of immunostaining [40] is very helpful. No findings about this were demonstrated in the PET-CT or TAC. CT and PET-CT showed no evidence of possible colonic metastases in our patient. If he had had a previous stool blood test, it might have advanced the diagnosis by performing an earlier colonoscopy. Fecal blood test, followed by early colonoscopy in positive cases, could potentially have a role in the staging of these patients. However, more research is needed to determine this.

In conclusion, we report a rare case of metastatic colonic carcinoma from the lung presenting at the emergency room with rectorrhagia. Gut metastasis from the lung are uncommon but we have to be aware of it in patients who present gastrointestinal symptoms.
